# Preharvest Nutrient Deprivation Reconfigures Nitrate, Mineral, and Phytochemical Content of Microgreens

**DOI:** 10.3390/foods10061333

**Published:** 2021-06-09

**Authors:** Marios C. Kyriacou, Christophe El-Nakhel, Georgios A. Soteriou, Giulia Graziani, Angelos Kyratzis, Chrystalla Antoniou, Alberto Ritieni, Stefania De Pascale, Youssef Rouphael

**Affiliations:** 1 Department of Vegetable Crops, Agricultural Research Institute, Nicosia 1516, Cyprus; m.kyriacou@ari.gov.cy (M.C.K.); soteriou@ari.gov.cy (G.A.S.); A.Kyratzis@ari.gov.cy (A.K.); chrystalla.antoniou@ari.gov.cy (C.A.); 2 Department of Agricultural Sciences, University of Naples Federico II, 80055 Portici, Italy; christophe.elnakhel@unina.it (C.E.-N.); depascal@unina.it (S.D.P.); 3 Department of Pharmacy, University of Naples Federico II, 80131 Naples, Italy; giulia.graziani@unina.it (G.G.); alberto.ritieni@unina.it (A.R.)

**Keywords:** antinutritive agents, carotenoids, bioactive value, flavonol glycosides, functional foods, hydroxycinnamic acids, polyphenols

## Abstract

While imparting gastronomic novelty and sensory delight, microgreens also constitute rudimentary leafy greens packed with nutrients and phytochemicals. As such, they comprise an upcoming class of functional foods. However, apart from bioactive secondary metabolites, microgreens also accumulate antinutritive agents such as nitrate, especially under conducive protected cultivation conditions. The current work examined nutrient deprivation before harvest (DBH), applied by replacing nutrient solution with osmotic water for six and twelve days, as a strategy for reducing microgreen nitrate levels in different species (lettuce, mustard, and rocket). The three species were sown on a peat-based substrate, cultivated in a controlled climate chamber, and harvested 18 days after sowing, when the first two true leaves emerged. DBH impact on major constituents of the secondary metabolome, mineral content, colorimetric, and yield traits was appraised. Nitrate and mineral content were determined through ion chromatography, phenolic composition through UHPLC-Q-Orbitrap HRMS, and carotenoid composition through HPLC-DAD. Nutrient deprivation was effective in reducing nitrate content; however, effective treatment duration differed between species and decline was more precipitous in nitrate hyperaccumulating species such as rocket. Quercetin and kaempferol glycosides were the flavonol glycosides most abundant in brassicaceous microgreens, whereas lettuce microgreens were steeped in caffeoyl quinic acid. DBH interacted with species as it increased the total phenolic content of lettuce, decreased that of rocket, but did not affect mustard. Further research to link changes in phenolic composition to the sensory and in vivo bioactive profile of microgreens is warranted. Notably, brief (≤6 days) DBH can be applied across species with moderate or no impact on the phenolic, carotenoid, and mineral composition of microgreens. Brief DBH applications also have limited impact on microgreens’ yield and colorimetric traits hence on the commercial value of the product. They can therefore be applied for reducing microgreen nitrate levels without significantly impacting key secondary metabolic constituents and their potential bioactive role.

## 1. Introduction

Microgreens upgrade the color and sensory palette of modern foods as reflected in the appealing adjectives they have been ascribed, which range from “vegetable confetti” [1,2,3,4], to “lingerie of the culinary world” [4], “functional foods”, “superfoods” [1,5], and much more. Undoubtedly, microgreens infuse human diets with gastronomic novelty [4]; moreover, they serve as dietary carriers of health-promoting plant secondary metabolites, while they are concomitantly renowned for their offbeat tastes, alluring colors, and subtle textures [3,4,6,7]. Microgreens have been commonly used for garnishing fancy dishes, salads and sandwiches [3,7,8], but lately they constitute basic ingredients of savory and sweet dishes with unconventional organoleptic profiles [5,8]. Noteworthy characteristics of microgreens include compact form, accelerated production, limited space requirement for growth, and high crop turnover, all of which render them an appealing specialty product in the modern horticultural supply chain and a crop well adapted to protected cultivation even in urban environments [4,7]. As stated by El-Nakhel et al. [9], protected cultivation, such as indoor growing modules, can produce fresh vegetables of constant quality owing to precise control of growth conditions yearlong.

Plant foods, including microgreen vegetables, are naturally biofortified with an array of bioactive compounds such as vitamins, minerals, and secondary metabolites [6,10]. Aside from desirable secondary metabolites however, vegetables can also accumulate antinutritive agents such as nitrate, especially under conducive protected cultivation conditions [10,11]. The metabolic products of nitrate have come under investigation for possible association with certain types of cancer and the methemoglobinemia syndrome [12]. Excessive accumulation of nitrate is frequently observed in leafy greens and poses a potential peril to human well-being [10,13]; therefore, tolerance levels for nitrate have been set (Regulation No. 1882/2006) by the European Commission for particular species under protected and open-field cultivation [14]. A cutback on the nitrate content of vegetable foods is therefore desirable in order to underscore their nutritive and nutraceutical value [15].

Nitrate uptake, assimilation, translocation, and accumulation in plants are driven by manifold internal (genetic variability, concerted gene expression and enzymatic activity, and ontogenetic stages) and external factors (N form, concentration, and application time; light intensity, quality, and photoperiod; air temperature; and CO_2_ concentration) that also influence the sensory and phytochemical traits of fresh vegetables [10,16,17]. Nitrate is the main form of nitrogen readily taken up by crops and is vital for protein and nucleic acid biosynthesis [13], but also vital for sustaining maximal yields [16]. However, excessive nitrate fertilization promotes nitrate accumulation in plant tissues [13,16], especially in petioles, leaves, and stems [15,16]. As stated by Colla et al. [16], variable preharvest approaches can be considered for lowering nitrate content in plants: (i) diminishing nitrate concentration in the fertigation solution, (ii) replacing nitrate with urea or ammonium, (iii) replacing the nutrient solution with water or a nitrate-free solution for a time period before harvest, (iv) replacing calcium nitrate with calcium chloride, (v) manipulating light spectral composition and intensity, and (vi) using low-accumulating genotypes.

Conventionally, microgreens have been regarded as low accumulators of nitrate among salad crops; however, relatively high concentrations of nitrate were detected in some microgreen species [7,18]. Moreover, one of the most popular families exploited for microgreens production is the *Brassicaceae*, which comprises nitrate hyperaccumulators such as rocket [1,16]. Few studies have dealt with fertigation strategies aimed to reduce nitrate accumulation in microgreens, of which we mention El-Nakhel et al. [6] and Pannico et al. [19] who adopted drastic strategies of fertigation with a quarter-strength (¼) nutrient solution (400 μS cm^−1^) or distilled water for the entire growth cycle, and Palmitessa et al. [5] who used ½, ¼, and ⅛ strength and different NH_4_:NO_3_ molar ratios. Nutrient deprivation strategies for a few days before harvesting, are known for eliciting changes in the plant secondary metabolome [9]. Similarly, the potential for eliciting the buildup of bioactive compounds by changing the associated enzymatic activity and gene expression was demonstrated in sprouts under appropriate conducive conditions [20]. Among numerous elicitors currently investigated on sprouts and microgreens, the most common are abiotic elicitors, which include seed priming with salts, mineral biofortification, and elicitation on mother plants transferred epigenetically to the sprouting seeds [21]. Biotic elicitors are another class that have attracted research interest, and these include fungal and yeast inoculation, and the inclusion of bacteria in substrates or seeds. Biostimulation is yet another upcoming area of interest that includes both biotic and abiotic factors as well as their combinatorial effects as elicitors. The most notable changes elicited relate to the products of the phenylpropanoid pathway. Previous studies have highlighted the antihypertensive [22] and anti-inflammatory effects [23] of plant-derived phenolics, the composition and concentration of which in vegetables is modulated by genetic, ontogenetic, environmental, and cultural factors [24]. Additionally, qualitative differences in phenolic composition were found critical in respect to lipid peroxidation and A549 cell proliferation [25]. Quercetin derivatives, for instance, were shown to exert pronounced antioxidant and anti-inflammatory activity and a protective role on other dietary antioxidants present in the human plasma [26,27].

Based on the above, it may be inferred that nutrient deprivation treatments before harvesting microgreens may potentially exert a dual effect: (a) reduce the concentration of nitrate and (b) modulate the composition and concentration of secondary metabolites that contribute to the bioactive value of microgreens. In order to examine these hypotheses, the current study appraised the response of three common microgreen species (lettuce, rocket, and mustard), grown in a strictly controlled climate chamber environment, to nutrient deprivation treatments of variable duration applied before harvest. Treatment effects were appraised in terms of yield traits, nitrate, and mineral content determined through ion chromatography, phenolic composition determined through UHPLC-Q-Orbitrap HRMS, and carotenoid composition determined through HPLC-DAD. The current study advances our understanding of the utility of nutrient deprivation strategies as a tool for modulating the qualitative attributes of microgreens and reducing their content in anti-nutrients like nitrate.

## 2. Materials and Methods

### 2.1. Genetic Material, Climate Chamber Setup, and Nutrient Solution Treatments

Three nitrate accumulating species from two different families (Brassicaceae and Asteraceae) were grown and harvested at the appearance of the second true leaf: mustard (*Brassica juncea* (L.) Czern cv. Osaka purple; Condor Seed Production, Yuma Arizona, USA), rocket (*Diplotaxis tenuifolia* cv. Wild Rocket, Napoli; CN Seeds Ltd., Pymoor, Ely, Cambrigeshire, UK), and lettuce (*Lactuca sativa* L. cv. Grand Rapids TBR; West Coast Seeds, Delta, British Columbia, Canada). The three species were sown respectively at densities of 60,000, 160,000, and 50,000 seeds m^−2^, and harvested 18 days after sowing (DAS), when the first two true leaves emerged. All microgreens were cultivated in a controlled climate chamber (MIR-554 growth chamber, Panasonic, Gunma, Japan) at the Department of Vegetable Crops of the Agricultural Research Institute (ARI) (Nicosia, Cyprus). Plastic trays (19 cm × 14 cm × 6 cm) were filled with a peat-based substrate (Floragard Vertriebs-GmbH, Oldenburg, Germany). Microgreens were subjected to three different fertigation strategies that consisted of nutrient solution termination at different days before harvest (DBH): 0, 6, and 12, to be replaced with osmotic water (pH = 6 ± 0.2 and EC = 150 ± 50 μS cm^−1^). The adopted nutrient solution was a quarter-strength modified Hoagland solution (pH = 6 ± 0.2 and EC = 500 ± 50 μS cm^−1^), described in detail in Kyriacou et al. [28].

The climate chamber was set at 24/18 ± 2 °C, day/night temperatures, corresponding to a photoperiod of 12 h, and a relative humidity in the range of 65–75 ± 5%. The artificial light of the climate chamber was provided by an LED panel (K5 Series XL 750, Kind LED, Santa Rosa, CA, USA), of which the channels were set at: 45% Red, 10% Green-Yellow, 45% Blue, offering a mean irradiance of 300 ± 15 μmol m^−2^ s^−1^ at canopy level, and wavelengths ranging between 400 and 700 nm while procuring an optimal absorption spectrum for photosynthesis. The trays were rotated during the growth cycle on a daily basis, to secure a homogenous repartition of light, humidity, and temperature over the different treatments across the climate chamber.

### 2.2. Colorimetric Measurement of Microgreens Canopy, Sampling, and Yield Assessment

Eighteen DAS, at the two-true-leaf stage, the CIELAB color space parameters (L*, a* and b*) were measured at the microgreens canopy level through a portable Minolta Chroma meter (CR-400, Minolta Co. Ltd., Osaka, Japan), then the hue angle (h°) and chroma (C*) were calculated. Each tray/replicate received eight measurements across its entire surface. Immediately after, all microgreens were harvested contemporarily by means of scissors by cutting the entire seedlings at substrate level; fresh weight was immediately assessed and expressed as kg fresh weight m^−2^. A sub-sample of each replicate was dried at 65 °C in a forced-air oven until reaching constant dry weight, which was used to calculate the dry matter (DM) percentage. This oven-dried subsample was ground and used for macro-minerals analysis, whereas the remaining part of each sample was stored at −80 °C and lyophilized prior to phytochemical analyses.

### 2.3. Analysis of Nitrate and Macro-Minerals by Ion Chromatography

The oven-dried material of microgreens was used to determine the content of nitrate and macro-minerals following a previously described methodology [29]. In brief, 250 mg of the desiccated microgreen tissue per sample were extracted in 50 mL of ultrapure water and immersed in a water bath (ShakeTemp SW 22, Julabo, Seelbach, Germany) for 10 min at 80 °C and constant shaking. Subsequently, the extracts were centrifuged and the supernatant was collected and stored in vials for chromatographic analysis using an ion chromatography system (ICS-3000, Dionex, Sunnyvale, CA, USA) coupled to an electrical conductivity detector. Nitrate and macro-minerals (phosphorus [P], potassium [K], calcium [Ca], magnesium [Mg], and sulfur [S]) content was determined on a dry weight basis (mg g^−1^) and then converted to mg kg^−1^ fresh weight, based on the corresponding DM content.

### 2.4. Analysis of Carotenoids by HPLC-DAD

Carotenoids were extracted from lyophilized microgreen samples, separated, and quantified as previously described by Kyriacou et al. [28] on an Agilent HPLC system (Agilent Technologies, Santa Clara, CA, USA) furnished with a 1200 Series quaternary pump and a 1260 Diode Array Detector Separation. Separation was accomplished with a Gemini C18 (Phenomenex, Torrance, CA, USA) reverse phase column (250 mm × 4.6 mm, 5 μm). Quantification using β-carotene and lutein commercial standards was performed on calibration curves established at six concentrations from 5 to 100 μg mL^−1^ and expressed in mg kg^−1^ DW.

### 2.5. Analysis of Polyphenols by UHPLC-Q-Orbitrap HRMS

Polyphenols were extracted from lyophilized micro-greens with methanol/water (60:40, v/v) and analyzed according to the method detailed in Kyriacou et al. [17]. Analysis was performed using a UHPLC system (Dionex UltiMate 3000, Thermo Fisher Scientific, Waltham, MA, USA) coupled to a Q-Exactive Orbitrap mass spectrometer (UHPLC, Thermo Fischer Sci-entific, Waltham, MA, USA). Polyphenols were separated using a Luna Omega PS 1.6 µm (50 mm × 2.1 mm, Phenomenex) column. Identification and quantification of compounds was accomplished using a Q-Exactive Orbitrap mass spectrometer (UHPLC, Thermo Fischer Scientific, Waltham, MA, USA) operated in fast negative/positive ion switching mode (Thermo Scientific, Bremen, Germany). Data analysis and processing were performed using software Xcalibur v. 3.0.63 (Xcalibur, Thermo Fisher Scientific, Waltham, MA, USA). Phenolic compounds were identified and quantified against calibration curves of available standards and expressed in µg g^−1^ DW. For standards that were not available, identification MS/MS experiments were employed.

### 2.6. Experimental Design and Statistical Analysis

A completely randomized experimental design was applied. Treatments were replicated three times with each replicate corresponding to a single tray of microgreens. Two-way analysis of variance (ANOVA) with species and nutrient deprivation as the main factors was performed using JMP statistical package (SAS Institute, Inc., Cary, NC, USA). Means were compared and separated according to the Tukey–Kramer HSD test.

## 3. Results

### 3.1. Yield Characteristics

Fresh yield was significantly affected by both species and DBH treatment, but variability was greater in response to species than it was between different DBH treatments (Table 1). The fresh yield of the highest yielding species (mustard) was 85.9% higher than that of the lowest yielding species (rocket). Fresh yield demonstrated a near-linear incremental decrease in response to DBH treatment duration. Increasing DBH from zero to six or from six to 12 days resulted in 5.6% and 11.8% decrease in fresh yield, respectively. Yield in terms of dry weight was significantly affected only by species, however a limited response to DBH was observed for rocket, the dry weight of which increased after 6-DBH and 12-DBH treatments. Dry matter content differed significantly between species with rocket demonstrating overall the highest DM and lettuce the lowest. Rocket was also the species most responsive to DBH with DM increasing with each DBH increment, whereas mustard incurred an increase in DM only at 12 DBH and lettuce no significant change in response to DBH.

### 3.2. Canopy Colorimetry

Genotype had a significant effect on all colorimetric attributes of the microgreen canopy determined in the CIELAB color space (Table 2). Nutrient deprivation treatments however did not have a horizontal effect on all colorimetric attributes. DBH treatments affected only the variables defining color intensity (a*, b* and chroma-C*) whereas their effect on the lightness (L*) and the quality of microgreens color denoted by hue angle (h°) was non-significant, with the exception of mustard that incurred a significant reduction in L* after 12-DBH treatment. Lettuce microgreens had the brightest canopy and mustard the darkest. Mustard microgreens had the lowest intensity of green and yellow color, expressed as the lowest negative values of color component a* and the lowest positive values of color component b*. However, the lowest overall color intensity (chroma) was demonstrated by rocket and the highest by lettuce. With respect to the DBH treatments, the most intensely green colored canopy was obtained from the control (zero DBH), which however did not differ significantly from the 6-DBH treatment. The 6-DBH and 12-DBH treatments also did not differ significantly, despite a trend in nominal values for declining greenness as the DBH increased from six to twelve days. The overall color intensity of microgreens (Chroma) did not change with nutrient deprivation for mustard and rocket but decreased significantly in lettuce when DBH was extended to 12 days.

### 3.3. Nitrate and Mineral Content

The nitrate content of microgreens was significantly affected by both genotype and DBH treatment; moreover, a significant genotype–DBH interaction was observed (Table 3). Mean nitrate content in rocket microgreens (1111 ± 342 μg g^−1^) was near threefold that of lettuce and mustard. Significant reduction in nitrate levels was observed with the DBH treatments. Across genotypes, mean nitrate content was reduced to 36.3% and 13.5% of the control after six and twelve days of DBH treatment, respectively. However, a variable response to DBH treatments was observed in the three studied genotypes, with rocket demonstrating the highest proportional reduction in nitrate content at both the six (−72.9%) and twelve-day treatments (−90.3%). Lettuce microgreens on the other hand, incurred a significant decrease in nitrate levels only after twelve days of DBH whereas mustard microgreens incurred a significant reduction after six days of DBH but no further reduction at twelve days. Phosphorus and potassium content varied with genotype significantly with highest P and lowest K found in mustard, as opposed to lettuce microgreens that combined the lowest levels of P and the highest levels of K. Both P and K declined with DBH treatment, although significant reduction in P was observed after six and twelve days of DBH whereas significant K reduction was demonstrated only after twelve days of DBH. Reduction in P content after six days of DBH was however practically limited as it accounted for only 14.6% of the control levels (zero DBH). Divalent cations Ca and Mg varied in concentration significantly between genotypes but their response to DBH treatments was non-significant. Finally, S content was overall highest in mustard and lowest in lettuce microgreens and declined with increasing DBH treatment duration. A significant genotype–DBH interaction was observed as lettuce incurred significant reduction in S content after 12 days of DBH treatment as opposed to the microgreens of the brassicaceous species mustard and rocket that incurred significant reduction after six days of DBH. 

### 3.4. Carotenoid Content

Lutein and β-carotene were the principal carotenoid components quantified in the three species of microgreens examined (Table 4). Cruciferous microgreens attained higher lutein content than lettuce microgreens, whereas β-carotene was lower in mustard than lettuce and rocket microgreens. Total carotenoids were most abundant in rocket and least abundant in lettuce microgreens. Both carotenoid molecules and total carotenoids were subject to species × DBH interaction. Lutein content in lettuce was non-responsive to DBH treatment, as opposed to mustard, wherein it increased after 12 days of DBH treatment, and rocket, wherein it decreased after 12 days of DBH treatment. A pronounced change in β-carotene content was observed only in rocket microgreens with declining content as DBH treatment increased. The total carotenoids content of lettuce microgreens was unaffected by DBH treatment whereas that of mustard microgreens declined significantly in response to 6-DBH but did not differ between the control and 12-DBH. Rocket microgreens, on the other hand, demonstrated the greatest response to nutrient deprivation, with 6-DBH and 12-DBH reducing the total carotenoids content by 13.0% and 27.3%, respectively.

### 3.5. Phenolic Composition

Eighteen polyphenols were identified and quantified by UHPLC-Q-Orbitrap HRMS in the three microgreen species subjected to nutrient deprivation treatments before harvest (Table 5).

As a proportion of their total phenolic content, the phenolic composition of the three species comprised mainly flavonol glycosides (12.18–54.7%), hydroxycinnamic acids and their derivatives (31.7–85.9%), and flavone glycosides (0.2–13.5%). Phenolic acids were most concentrated in lettuce microgreens and least concentrated in mustard microgreens. Conversely, mustard microgreens were most abundant in flavonol and flavone glycosides. Total phenolic content ranged 715.5–1238.0 μg g^−1^ DW across species, being highest in lettuce and lowest in mustard microgreens, whereas a more limited range was encountered between DBH treatments (960.3–1053.4 μg g^−1^ DW). Variation in the phenolic components quantified and in the total phenolic content was determined principally by species and only moderately by the DBH treatments. The relative abundance of individual phenolic components varied widely among species. The most pronounced species differentiation was observed for caffeoyl quinic acid, which was at minimal levels (0.48–1.69 μg g^−1^ DW) in brassicaceous microgreens and at maximal levels (1055.3 μg g^−1^ DW) in lettuce microgreens, while similar variation was observed for quercetin-3-glucoside. Conversely, quercetin-3-sinapoyl triglucoside and synapoyl-hexose were found at their highest concentration in rocket microgreens and moderately concentrated in mustard microgreens but were, respectively, non-detectible and at minimal levels in lettuce microgreens.

The phenolic components affected by DBH treatments, as well as the total phenolic content of microgreens, also incurred significant species × DBH interaction that reflects a species-dependent response pattern to DBH treatments. (Table 5). Prominent examples of this interaction were as follows: caffeoyl quinic acid and quercetin-3-glucoside that climaxed after 6-DBH and 12-DBH treatments, respectively, in lettuce but remained unaltered in the rest species, ferulic acid that declined progressively with DBH duration in mustard but was unaltered in the rest species, and synapoyl-hexose that declined progressively with DBH duration in rocket but was unaltered in the rest of the species. An interaction was also observed with respect to quercetin-3-sinapoyl triglucoside that declined with DBH treatment in rocket, increased in mustard, and remained unaltered in lettuce. Interaction was also manifested in respect to the total phenolic content, which declined with DBH treatment in rocket, increased in lettuce and remained unaltered in mustard microgreens.

## 4. Discussion

### 4.1. Yield Characteristics

The lower yielding capacity of rocket microgreens presently demonstrated in comparison to lettuce and mustard microgreens, confirms similar findings from previous studies on the sensitivity of this species to nutrient deprivation [6,30]. Variable reduction in fresh yield when grown under complete deprivation of nutrient supplementation (i.e., irrigated only with distilled water), compared to the fertigated control, was demonstrated with microgreens of different brassicaceous species in the study of El Nakhel et al. [6]; wherein yield reduction ranged from 7.9% in cabbage to 47.4% in rocket. Analogous species-dependent yield reduction was also reported for different *Brassica* microgreens in response to variable nutrient solution strength [5]. In the present study however, nutrient deprivation limited to 6-DBH and 12-DBH did not interact with species for microgreens yield, which responded inversely to the extension of treatment duration similarly in all species. El Nakhel et al. [6] also reported a species × fertigation interaction for DM, ranging from no change in cabbage microgreens incurring to 9.8% and 26.8% increase in the DM of Brussel sprouts and rocket microgreens, respectively. The current study demonstrated similar interaction for DM ranging from no change in lettuce microgreens to 29.4% increase in rocket microgreens, underscoring the higher responsiveness of rocket microgreens to nutrient deprivation.

The present results indicate that microgreens fresh yield was dictated primarily by genotype. The deprivation of nitrogen and mineral supply before harvest had a detrimental effect on fresh yield, however the application of a brief six-day DBH treatment was demonstrated to have a moderate impact, which minimizes economic loss when applied. Moreover, the efficacy of applying brief DBH treatments to increase the DM content of microgreens is promising as a method of potentially enhancing their sensory and phytochemical profile [31]. The genotype-dependent response for DM presently demonstrated warrants however further research into the nature of this interaction before commercial production is targeted on responsive species.

### 4.2. Canopy Colorimetry

Colorimetric attributes of horticultural products arguably constitute indirect measurements of quality, particularly organoleptic quality and, to an extent, phytochemical content [32]. Visual quality is a fundamental constituent of the current regulatory context that defines crop-specific quality standards for horticultural products; moreover, consumers have been increasingly conditioned in perceiving visual traits as being indicative of sensory and functional quality [33]. The perceived quality of microgreens is strongly related to color since they have been established as garnishes in high gastronomy [1]. From the current study, it may be inferred that genotype is the main determinant of microgreens’ basic color attributes, especially lightness (or darkness) and hue. Notwithstanding their brief growth cycle, nutrient deprivation might potentially undermine color development in microgreens. In terms of overall color intensity (chroma), the current study shows that this effect is not consistent across genotypes, and its severity depends on DBH duration, having been manifested only in lettuce subjected to 12-DBH nutrient deprivation. Similarly, El Nakhel et al. [6] reported the absence of fertigation effect on microgreens L* and hue color attributes, and a species × fertigation interaction for chroma with Brussels sprouts and cabbage microgreens incurring non-significant changes as opposed to rocket that declined in chroma by 17.7% when fertigation was replaced by distilled water. In the current study however, loss of greenness (decreasing negative a* values) was registered in all species after 12-DBH nutrient deprivation. It may be inferred therefore that brief nutrient deprivation treatments (<6 days) can be applied without impacting microgreens’ color intensity and the commercial value of the product.

### 4.3. Nitrate and Mineral Content

The chief source of nitrate in the human diet is the consumption of vegetables, particularly leafy greens, of which rocket, lettuce, and spinach combine conduciveness to nitrate accumulation with high consumption, thus their maximum nitrate content is stipulated in the European Commission Regulation No 1882/2006 [14] (EC, 2006). Although nitrate in itself constitutes a relatively low health hazard, its reaction products and metabolites (nitrite, nitric oxide, and N-nitroso compounds) have been implicated in certain types of oncogenesis and the methemoglobinemia syndrome [12]. Microgreens are generally consumed in limited quantity but nonetheless classify as leafy greens that contribute to the human diet in various forms, from garnishes to smoothies, and their most common genotypes derive from the Brassicaceae, which includes known nitrate hyperaccumulators such as rocket [1,16]. Moreover, certain wild genotypes (e.g., small burnet (*Sanguisorba minor*), wild mustard (*Sinapis arvensis*), and common dandelion (*Taraxacum officinale*)) that comprise a repository of interest for the growing microgreens industry, have in previous studies demonstrated nitrate hyper-accumulating capacity with concentrations ranging from 2834 to 7129 mg kg^−1^ [18].

In the current study, rocket microgreens attained threefold higher nitrate concentration than lettuce and mustard. The latter two attained similar nitrate content as previously found in coriander, kohlrabi, pak choi, basil, beet, and rapini microgreens sustained with nutrient solutions on various substrates [34,35,36]. The detected levels were in all cases, including rocket microgreens, well below the tolerance maxima stipulated in EC Reg. No 1882/2006 [14]. Our current results moreover corroborate previous studies that demonstrated a comparably lower accumulation of nitrate in microgreens than their mature counterparts [37,38,39]. The nutrient deprivation treatment was demonstrated overall as effective in reducing the nitrate content of all genotypes studied. Analogous reduction of leaf nitrates was found in hydroponically cultivated cardoon leaves subjected to 15 day nutrient deprivation prior to harvest [40]. The duration of the nutrient deprivation treatment required for effective reduction of nitrate levels differed between genotypes, with the nitrate hyperaccumulating species, such as rocket, being more responsive to DBH treatments than other species. It is worth reiterating that rocket microgreens not deprived of fertigation attained 3.6- and 4.9-fold higher nitrate content than mustard and lettuce, respectively; however, six- and twelve-day nutrient deprivation resulted in dramatic reduction on nitrate by 72.9% and 90.3%, respectively. By decreasing the strength of Hoagland solution, El Nakhel et al. [9] observed a similar genotype-dependent response in the total N content of green vs. red Salanova lettuce, with the content remaining unaltered in the former but decreasing in the latter. As presently demonstrated across species, a brief six-day nutrient deprivation constitutes a significant tool for depleting nitrate deposits prior to harvesting microgreens, thus increasing food safety in terms of antinutritive agents such as nitrate.

The role of minerals in human nutrition is critical in supporting optimum development while maintaining body homeostasis and metabolic functionality, as well as in preventing nutritional deficiencies associated with physiological disorders [41]. Fruits and vegetables contribute substantially as dietary sources of minerals, with their percentage contribution towards K, Na, Ca, Mg, and P dietary requirements accounting, respectively, for 35, 11, 7, 24, and 11% of total requirements [42]. Analytical data on the mineral composition of microgreens are yet limited but the role of genotype as key determinant of mineral composition has been highlighted and is reiterated by the current results. Notwithstanding genotype differences, the current work corroborates previous studies that reported microgreen mineral content in declining order as K > Ca > Mg > S [5,17,43]. However, data comparison between studies is hampered by variation in experimental conditions that may effectively modulate the mineral content of microgreens such as, nutrient solution composition, light quality, intensity and photoperiod, growth substrate, and harvest maturity [1].

The present work highlighted that the P and K contents of microgreens are highly responsive to nutrient deprivation across genotypes with both minerals having demonstrated moderate reduction, as opposed to the divalent ions Ca^++^ and Mg^++^ that were non-responsive. This outcome might be attributed to the mineral composition of the peat-based substrate that apparently included adequate levels of Ca^++^ and Mg^++^ to support the brief growth cycle of microgreens under nutrient deprivation conditions. We have previously demonstrated that natural fiber and synthetic substrates differing in physicochemical constitution may modulate the mineral, and indirectly, the phytochemical composition of microgreens [36]. Moreover, it has been demonstrated that nutrient composition of typical peat-based substrates may support satisfactory growth of microgreens without supplying additional nutrients, which renders their cultivation readily accessible to non-professionals [19]. Considering that microgreens are marketed not only as flavorful and colorful condiments or garnishes but also as potent sources of phytochemicals and minerals, the impact of depriving nutrients (solution- or substrate-provided) on the mineral content of microgreens is presently underscored. Regarding the genotype-dependent response to nutrient deprivation observed for S content, the higher responsiveness of brassicaceous species most likely relates to their increased demands for S implicated in glucosinolate biosynthesis [44].

### 4.4. Carotenoid Content

The content of lutein and β-carotene, the major carotenoid compounds found in microgreens, contributes to their bioactive value as they constitute lipophilic antioxidants owing to the light-absorbing and ROS-quenching properties of their polyene chains [45]. Lutein dietary uptake or supplementation has been linked to protection against oxidative damage and macular degeneration, while β-carotene is an essential precursor of vitamin A required for development and effective function of the immune and optical system [46]. Based on the results of the current study, we can conclude that carotenoid levels in microgreens are primarily dictated by genotype, which supports previous studies on the carotenoid content of microgreens of different species or cultivars grown under variable conditions [34,36,38,47]. Moreover, response to nutrient deprivation with respect to carotenoid composition is genotype-specific, with certain species, such as lettuce, being non-responsive and others, such as rocket, being depleted of carotenoid content with increasing treatment duration. Genotype-dependent decline in carotenoids was also reported in lettuce grown hydroponically under decreasing concentrations of nutrient solution [9]. It seems plausible that depletion of carotenoids with nutrient deprivation is more pronounced in carotenoid-rich species. However, even in carotenoid-rich species the depletion of carotenoids during the first six days of DBH treatment seems moderate, with 14.9% and 37.6% reduction taking place between days 0–6 and 6–12, respectively. Therefore, brief DBH treatments are likely to cause moderate depletion of carotenoids in carotenoid-rich species, which arguably would not compromise significantly the bioactive value of the product.

### 4.5. Phenolic Composition

The phenolic composition of microgreens appraised through UHPLC-Q-Orbitrap HRMS was dominated by flavonol glycosides in mustard and by hydroxycinnamic acids in lettuce and rocket. Quercetin and kaempferol glycosides were the flavonol glycosides most abundant in brassicaceous microgreens, whereas lettuce microgreens were steeped in caffeoyl quinic acid. The current findings largely corroborate previous reports on microgreens’ composition and confirm the wide variation in phenolic components encountered even among related brassicaceous genotypes [48,49,50]. It is further demonstrated that nutrient deprivation before harvest may impact significantly the phenolic constituents of microgreens and their total phenolic content. However, the effect of DBH treatment on most phenolic components and on the total phenolic content seems highly species-dependent. Intriguingly, lettuce microgreens were the most positively responsive to nutrient deprivation, as both 6-DBH and 12-DBH treatments increased the total flavonol glycosides, total hydroxycinnamic acids, and the total phenolic contents. Mustard microgreens were moderately responsive, as their flavonol glycoside increased in response to 6-DBH and 12-DBH treatments but not their hydroxycinnamic acids, and total phenolic contents. Finally, rocket microgreens were the most negatively responsive to nutrient deprivation, which resulted in the reduction of their content in flavonol glycosides, hydroxycinnamic acids and total phenols. It is therefore apparent that aside from its utility in reducing the nitrate levels of microgreens, DBH treatments mimic eustress applications in modulating the products of the phenylpropanoid pathway [9], particularly when they extend beyond 6-DBH. The positive response to DBH treatments exhibited by certain species, such as lettuce, which incurred significant increase in its flavonol glycoside and hydroxycinnamic acid components, might render nutrient deprivation an important tool for enhancing the bioactive value of microgreens. This prospect is encouraging in light of previous findings concerning the analgesic and anti-inflammatory activities of hydroxycinnamic acids [51], and the in vitro antiproliferative, and in vivo antioxidant and anti-inflammatory activity of quercetin derivatives [23,24]. Such applications, however, warrant further research to determine the responsiveness of targeted species to nutrient deprivation treatments, as well as to link changes in phenolic composition to variation in the sensory and in vivo bioactive value of microgreens [52]. It is nonetheless noteworthy that a brief six-day nutrient deprivation before harvest has had overall a moderate impact or no effect on the phenolic composition of microgreens across species; thus, it can be applied to reduce nitrate residual in microgreens without dramatically shifting the phenolic composition and its potential implications for the bioactive value.

## 5. Conclusions

Nutrient deprivation was an effective method for reducing the nitrate content of microgreens, however effective treatment duration differed between species. Decline in nitrate content is more precipitous in hyperaccumulating species such as rocket, which incurred 72.9% and 90.3% reduction of nitrate content after 6-DBH and 12-DBH treatments, respectively. The present work also demonstrated that brief (≤6 days) nutrient deprivation treatment before harvest can be applied across species with moderate or no impact on the phenolic, carotenoid, and mineral composition of microgreens. Such brief DBH applications also seem to have limited impact on microgreens’ yield and color intensity and therefore on the commercial value of the product. It can therefore be applied to reduce nitrate levels in microgreens without dramatically shifting their key secondary metabolite content and its potential implications for bioactive value.

## Figures and Tables

**Table 1:** Yield attributes of three microgreen species (lettuce, mustard, and rocket) in response to nutrient deprivation treatment (DBH) applied for 6 or 12 days before harvest. All data are expressed as mean ± standard error, *n* = 3.

	Fresh Weight		Dry Weight		DM	
	(kg m^−2^)		(g m^−2^)		(%)	
Species	***		***		***	
DBH	***		n.s.		***	
Species*DBH	n.s.		***		***	
Lettuce	2.35 ± 0.06	b	106.25 ± 1.39	b	4.53 ± 0.08	c
Mustard	2.68 ± 0.07	a	162.18 ± 1.38	a	6.08 ± 0.13	b
Rocket	1.44 ± 0.05	c	100.71 ± 2.27	c	7.09 ± 0.4	a
0	2.33 ± 0.2	a	122.28 ± 11.11		5.31 ± 0.27	c
6	2.2 ± 0.18	b	124.03 ± 9.67		5.76 ± 0.34	b
12	1.94 ± 0.18	c	122.83 ± 9		6.63 ± 0.55	a
Lettuce, 0	2.53 ± 0.04		108.74 ± 0.91	b	4.3 ± 0.04	d
Lettuce, 6	2.38 ± 0.01		107.4 ± 1.29	b	4.51 ± 0.04	d
Lettuce, 12	2.14 ± 0.06		102.6 ± 3.23	b	4.79 ± 0.07	d
Mustard, 0	2.89 ± 0.03		165.62 ± 2.04	a	5.73 ± 0.12	c
Mustard, 6	2.72 ± 0.03		162.52 ± 1.2	a	5.98 ± 0.02	c
Mustard, 12	2.43 ± 0.03		158.4 ± 1.99	a	6.52 ± 0.11	b
Rocket, 0	1.57 ± 0.05		92.47 ± 0.36	c	5.91 ± 0.21	c
Rocket, 6	1.5 ± 0.02		102.17 ± 1.38	b	6.81 ± 0.01	b
Rocket, 12	1.26 ± 0.03		107.49 ± 1.42	b	8.57 ± 0.11	a

n.s., *** Nonsignificant or significant at *p* ≤ 0.001, respectively. Different letters within each column indicate significant differences according to Tukey–Kramer HSD test (*p* = 0.05). DM: dry matter percentage.

**Table 2:** Canopy colorimetric attributes of three microgreen species (lettuce, mustard, rocket) in response to nutrient deprivation treatment (DBH) applied for 6 or 12 days before harvest. All data are expressed as mean ± standard error, *n* = 3.

	L*		a*		b*		Chroma		HUE	
	(0–100)		(−60/+60)		(−60/+60)		√(a^2^ + b^2^)		(0–360)°	
Species	***		***		***		***		***	
DBH	n.s.		**		**		**		n.s.	
Species*DBH	**		n.s.		n.s.		*		n.s.	
Lettuce	45.29 ± 1.76	a	−8.59 ± 0.24	b	28.73 ± 0.74	a	29.99 ± 0.78	a	106.65 ± 0.18	b
Mustard	31.88 ± 1.15	c	−6.15 ± 0.26	a	21.96 ± 0.42	c	22.81 ± 0.46	b	105.6 ± 0.46	b
Rocket	37.9 ± 0.63	b	−8.42 ± 0.1	b	24.46 ± 0.36	b	13.5 ± 0.15	c	109.03 ± 0.29	a
0	38.47 ± 3.18		−8.15 ± 0.47	b	26.02 ± 1.3	a	23.16 ± 2.73	a	107.42 ± 0.73	
6	39.17 ± 1.91		−7.78 ± 0.37	ab	25.19 ± 0.98	ab	22.24 ± 2.4	a	107.16 ± 0.54	
12	37.44 ± 1.47		−7.23 ± 0.43	a	23.95 ± 0.94	b	20.9 ± 2.1	b	106.7 ± 0.5	
Lettuce, 0	50.17 ± 0.39	a	−9.29 ± 0.07		30.91 ± 0.21		32.28 ± 0.22	a	106.73 ± 0.1	
Lettuce, 6	45.2 ± 2.09	ab	−8.52 ± 0.31		28.78 ± 0.24		30.01 ± 0.28	ab	106.48 ± 0.52	
Lettuce, 12	40.5 ± 3.05	bc	−7.96 ± 0.37		26.51 ± 1.3		27.68 ± 1.34	b	106.73 ± 0.32	
Mustard, 0	28.66 ± 0.7	d	−6.5 ± 0.72		22.82 ± 0.58		23.74 ± 0.72	c	105.84 ± 1.4	
Mustard, 6	33.6 ± 2.33	cd	−6.35 ± 0.1		22.29 ± 0.64		23.17 ± 0.63	c	105.91 ± 0.38	
Mustard, 12	33.39 ± 1.44	cd	−5.59 ± 0.28		20.78 ± 0.49		21.52 ± 0.53	c	105.05 ± 0.47	
Rocket, 0	36.56 ± 1.52	cd	−8.67 ± 0.13		24.32 ± 1.16		13.47 ± 0.47	d	109.68 ± 0.58	
Rocket, 6	38.71 ± 0.32	bc	−8.48 ± 0.17		24.52 ± 0.3		13.53 ± 0.11	d	109.1 ± 0.46	
Rocket, 12	38.44 ± 1.01	bc	−8.12 ± 0.06		24.55 ± 0.38		13.49 ± 0.16	d	108.32 ± 0.16	

n.s., *, **, *** Nonsignificant or significant at *p* ≤ 0.05, 0.01, and 0.001, respectively. Different letters within each column indicate significant differences according to Tukey–Kramer HSD test (*p* = 0.05).

**Table 3:** Nitrate and mineral content of three microgreen species (lettuce, mustard, and rocket) in response to nutrient deprivation treatment (DBH) applied for 6 or 12 days before harvest. All data are expressed as mean ± standard error, *n* = 3.

Source of Variance	Nitrate		P		K		Ca		Mg		S	
	(mg kg^−1^ FW)		(mg g^−1^ DW)		(mg g^−1^ DW)		(mg g^−1^ DW)		(mg g^−1^ DW)		(mg g^−1^ DW)	
Species	***		***		***		***		***		***	
DBH	***		***		***		n.s.		n.s.		***	
Species*DBH	***		n.s.		n.s.		*		*		***	
Lettuce	313.36 ± 53.52	b	3.55 ± 0.13	c	97.68 ± 1.11	a	14.66 ± 0.39	c	7.75 ± 0.13	b	3.01 ± 0.16	c
Mustard	386.81 ± 82.58	b	4.97 ± 0.14	a	69.57 ± 1.47	c	21.32 ± 0.46	a	9.90 ± 0.20	a	4.39 ± 0.37	a
Rocket	1111.22 ± 341.57	a	4.34 ± 0.21	b	77.78 ± 1.11	b	19.26 ± 0.33	b	7.91 ± 0.18	b	4.08 ± 0.45	b
0	1208.73 ± 313.05	a	4.86 ± 0.23	a	84.35 ± 4.34	a	18.48 ± 0.85		8.35 ± 0.28		4.99 ± 0.38	a
6	439.25 ± 56.77	b	4.15 ± 0.19	b	82.2 ± 4.21	a	18.42 ± 1.00		8.43 ± 0.38		3.66 ± 0.17	b
12	163.40 ± 19.30	c	3.86 ± 0.22	c	78.47 ± 4.26	b	18.34 ± 1.28		8.79 ± 0.46		2.84 ± 0.14	c
Lettuce, 0	502.98 ± 9.23	bcd	3.99 ± 0.06		101.24 ± 1.54		15.22 ± 0.48	d	7.99 ± 0.02	c	3.48 ± 0.05	bcd
Lettuce, 6	300.83 ± 17.81	de	3.54 ± 0.06		97.41 ± 0.69		14.69 ± 0.40	d	7.76 ± 0.32	c	3.13 ± 0.06	cde
Lettuce, 12	136.27 ± 15.18	e	3.12 ± 0.09		94.39 ± 0.5		14.07 ± 1.03	d	7.52 ± 0.19	c	2.42 ± 0.04	e
Mustard, 0	686.13 ± 18.91	b	5.45 ± 0.14		74.19 ± 1.17		20.15 ± 0.63	abc	9.38 ± 0.29	ab	5.72 ± 0.09	a
Mustard, 6	356.09 ± 8.58	cde	4.84 ± 0.15		68.7 ± 1.97		21.21 ± 0.65	ab	9.84 ± 0.18	a	4.17 ± 0.25	b
Mustard, 12	118.2 ± 7.44	e	4.63 ± 0.08		65.81 ± 1.64		22.59 ± 0.41	a	10.49 ± 0.21	a	3.28 ± 0.16	cd
Rocket, 0	2437.09 ± 188.52	a	5.14 ± 0.18		77.63 ± 2.29		20.06 ± 0.37	abc	7.68 ± 0.18	c	5.76 ± 0.22	a
Rocket, 6	660.84 ± 26.44	bc	4.07 ± 0.05		80.5 ± 0.67		19.36 ± 0.30	bc	7.69 ± 0.34	c	3.67 ± 0.15	bc
Rocket, 12	235.74 ± 13.41	de	3.82 ± 0.12		75.21 ± 1.49		18.35 ± 0.55	c	8.35 ± 0.27	bc	2.81 ± 0.14	de

n.s., *, *** Nonsignificant or significant at *p* ≤ 0.05 and 0.001, respectively. Different letters within each column indicate significant differences according to Tukey–Kramer HSD test (*p* = 0.05).

**Table 4:** Carotenoid content of three microgreen species (lettuce, mustard, and rocket) in response to nutrient deprivation treatment (DBH) applied for 6 or 12 days before harvest. All data are expressed as mean ± standard error, *n* = 3.

Source of Variance	Lutein		β-Carotene		Total Carotenoids	
	(mg kg^−1^ DW)		(mg kg^−1^ DW)		(mg kg^−1^ DW)	
Species	***		***		***	
DBH	n.s.		***		***	
Species*DBH	***		***		***	
Lettuce	303.05 ± 3.51	b	442.48 ± 5.31	a	745.53 ± 7.28	b
Mustard	395.71 ± 20.31	a	263.24 ± 16.94	b	658.96 ± 34.23	c
Rocket	405.62 ± 21.98	a	458.42 ± 38	a	864.04 ± 57.8	a
0	381.7 ± 24.42		447.93 ± 41.59	a	829.63 ± 54.6	a
6	354.42 ± 20.89		363.7 ± 41.95	b	718.12 ± 53.95	b
12	368.26 ± 25.09		352.51 ± 22.94	b	720.77 ± 17.68	b
Lettuce, 0	303.11 ± 4.34	d	459.55 ± 6.08	b	762.66 ± 4.24	c
Lettuce, 6	297.85 ± 5.69	d	426.91 ± 2.65	b	724.76 ± 8.09	cd
Lettuce, 12	308.19 ± 8.37	d	440.97 ± 5.17	b	749.16 ± 13.54	c
Mustard, 0	390.56 ± 12.81	bc	299.86 ± 13.31	c	690.42 ± 5.48	cd
Mustard, 6	330.65 ± 4.28	cd	197.74 ± 3.97	d	528.39 ± 3.38	e
Mustard, 12	465.93 ± 13.21	a	292.13 ± 4	c	758.06 ± 9.39	c
Rocket, 0	451.44 ± 37.62	ab	584.38 ± 14.09	a	1035.82 ± 50.3	a
Rocket, 6	434.75 ± 8.69	ab	466.45 ± 6.79	b	901.2 ± 8.06	b
Rocket, 12	330.67 ± 5.13	cd	324.42 ± 11.95	c	655.1 ± 15.06	d

n.s., *** Nonsignificant or significant at *p* ≤ 0.001, respectively. Different letters within each column indicate significant differences according to Tukey–Kramer HSD test (*p* = 0.05).

**Table 5:** Phenolic composition of three microgreen species (lettuce, mustard, and rocket) in response to nutrient deprivation treatment (DBH) applied for 6 or 12 days before harvest. All data are expressed as mean ± standard error, *n* = 3.

**Flavonol Glycosides**																							
**Source of Variance**	**Kaempferol-3-Hydroxyferuloyl-Sophorotrioside-7-Glucoside**		**Quercetin-3-Sophoroside-7-Glucoside**		**Kaempferol-3-Glucoside**		**Kaempferol 3-Diglucoside**		**Kaempferol-3-Sinapoyl-Sophoroside-7-Glucoside**		**Kaempferol-3-Sinapoyl-Sophorotrioside-7-Glucoside**	**Quercetin-3-Sinapoyl Triglucoside**		**Quercetin-3-O-Rutinoside (Rutin)**		**Quercetin-3-Glucoside**		**Coumaroyl-Diglucoside**		**Isorhamnetin-3-Gentiobioside**		**Total Flavonol Glycosides**	
	**(μg g^−1^ DW)**		**(μg g^−1^ DW)**		**(μg g−1 DW)**		**(μg g^−1^ DW)**		**(μg g^−1^ DW)**		**(μg g^−1^ DW)**	**(μg g^−1^ DW)**		**(μg g^−1^ DW)**		**(μg g^−1^ DW)**		**(μg g^−1^ DW)**		**(μg g^−1^ DW)**		**(μg g^−1^ DW)**	
Species	***		***		***		***		***			***		***		***		***		***		***	
DBH	n.s.		n.s.		n.s.		n.s.		*		n.s.	***		n.s.		***		***		**		***	
Species*DBH	n.s.		***		n.s.		***		n.s.			***		***		***		**		n.s.		***	
Lettuce	n.d.		n.d.		5.58 ± 0.13	a	11.15 ± 0.67	b	n.d.		n.d.	n.d.		16.23 ± 0.89	b	124.7 ± 10.36	a	0.63 ± 0.06	b	n.d.		158.29 ± 11.56	c
Mustard	46.3 ± 0.78	a	10.53 ± 0.56	b	1 ± 0.09	b	18.57 ± 0.39	a	78.46 ± 1.03	a	79.96 ± 1.85	105.1 ± 8.74	b	24.03 ± 0.47	a	0.55 ± 0.08	b	1.54 ± 0.06	a	25.64 ± 1.97	a	391.68 ± 11.54	a
Rocket	0.19 ± 0.02	b	13.8 ± 0.56	a	n.d.		6.91 ± 0.36	c	0.65 ± 0.05	b	n.d.	321.21 ± 17.24	a	13.67 ± 0.85	c	3.04 ± 0.21	b	1.69 ± 0.14	a	8.76 ± 0.41	b	369.92 ± 18.33	b
0	22.86 ± 10.17		12.4 ± 1.59		3.28 ± 1.03		12.01 ± 1.92		38.86 ± 17.07	a	74.7 ± 1.84	216.18 ± 64.46	b	17.86 ± 1.97		29.91 ± 14.36	c	1.08 ± 0.13	b	15.67 ± 3.57	b	291.93 ± 44.52	b
6	22.91 ± 10.15		12.27 ± 0.47		3.21 ± 0.98		12.14 ± 1.6		41.16 ± 18.09	a	80.04 ± 2.13	237.86 ± 50.43	a	18 ± 1.29		44.88 ± 21.44	b	1.2 ± 0.19	b	21 ± 5.11	a	328.51 ± 42.17	a
12	23.96 ± 10.66		11.83 ± 0.55		3.39 ± 1.09		12.48 ± 1.78		38.64 ± 17.07	a	85.13 ± 2.39	185.42 ± 30.43	c	18.08 ± 1.86		53.5 ± 25.78	a	1.58 ± 0.21	a	14.92 ± 3.04	b	299.45 ± 30.08	b
Lettuce, 0	n.d.		n.d.		5.57 ± 0.24	a	9.83 ± 0.66	c	n.d.		n.d.	n.d.		14.52 ± 0.61	de	87.16 ± 4.15	c	0.59 ± 0.02	d	n.d.		117.66 ± 2.93	f
Lettuce, 6	n.d.		n.d.		5.37 ± 0.18	a	9.99 ± 0.09	c	n.d.		n.d.	n.d.		14.5 ± 0.18	de	130.42 ± 5.21	b	0.48 ± 0.12	d	n.d.		160.77 ± 5.01	e
Lettuce, 12	n.d.		n.d.		5.81 ± 0.24	a	13.63 ± 0.31	b	n.d.		n.d.	n.d.		19.67 ± 0.42	c	156.52 ± 3.93	a	0.81 ± 0.08	d	n.d.		196.44 ± 3.64	d
Mustard, 0	45.58 ± 1.04		8.88 ± 0.56	c	1 ± 0.14	b	19.4 ± 0.64	a	77.01 ± 1.14		74.7 ± 1.84	72.35 ± 6.17	d	25.57 ± 0.72	a	0.3 ± 0.01	d	1.32 ± 0.06	c	23.57 ± 1.05	b	349.68 ± 8.18	b
Mustard, 6	45.56 ± 1.59		11.78 ± 0.85	b	1.06 ± 0.25	b	18.36 ± 0.88	a	81.57 ± 1.93		80.04 ± 2.13	125.37 ± 7.24	c	22.97 ± 0.56	b	0.77 ± 0.13	d	1.59 ± 0.03	bc	31.83 ± 3.64	a	420.9 ± 11.28	a
Mustard, 12	47.76 ± 1.45		10.92 ± 0.69	bc	0.96 ± 0.07	b	17.95 ± 0.34	a	76.8 ± 0.62		85.13 ± 2.39	117.57 ± 2.67	c	23.56 ± 0.12	ab	0.57 ± 0.07	d	1.72 ± 0.07	b	21.51 ± 1.63	b	404.47 ± 3.49	a
Rocket, 0	0.14 ± 0.02		15.91 ± 0.14	a	n.d.		6.81 ± 0.44	d	0.71 ± 0.05		n.d.	360 ± 7.26	a	13.5 ± 0.95	ef	2.28 ± 0.09	d	1.34 ± 0.07	bc	7.77 ± 0.49	c	408.46 ± 8.09	a
Rocket, 6	0.26 ± 0.03		12.75 ± 0.35	b	n.d.		8.07 ± 0.19	cd	0.75 ± 0.09		n.d.	350.36 ± 2.98	a	16.52 ± 0.24	d	3.44 ± 0.12	d	1.53 ± 0.13	bc	10.17 ± 0.29	c	403.85 ± 2.99	a
Rocket, 12	0.16 ± 0.02		12.74 ± 0.48	b	n.d.		5.87 ± 0.36	d	0.48 ± 0.01		n.d.	253.26 ± 4.53	b	10.99 ± 0.31	f	3.4 ± 0.23	d	2.21 ± 0.07	a	8.33 ± 0.37	c	297.45 ± 3.37	c
**Hydroxycinnamic acids and derivatives**													**Flavone glycosides**										
**Source of variance**	**Synapoyl-hexose**		**Ferulic acid**		**Trisinapoyl-gentiobiose**		**Disinapoyl-gentiobiose**		**Caffeoyl quinic acid**		**Total hydroxycinnamic acids and derivatives**			**Apigenin-7-O-glucoside**		**Luteolin-7-O-glucoside**		**Total flavone glycosides**		**Total polyphenols**			
	**(μg g−1 DW)**		**(μg g−1 DW)**		**(μg g−1 DW)**		**(μg g−1 DW)**		**(μg g−1 DW)**		**(μg g−1 DW)**			**(μg g−1 DW)**		**(μg g−1 DW)**		**(μg g−1 DW)**		**(μg g−1 DW)**			
Species	***		***		***		***		***		***			***		***		***		***			
DBH	***		***		***		n.s.		***		**			n.s.		n.s.		n.s.		***			
Species*DBH	***		***		***		n.s.		***		***			n.s.		n.s.		n.s.		***			
Lettuce	5.35 ± 0.33	c	2.5 ± 0.19	b	n.d.		n.d.		1055.3 ± 31.9	a	1063.15 ± 32.13		a	n.d.		16.51 ± 0.36	b	16.51 ± 0.36	b	1237.95 ± 39.5			a
Mustard	96.86 ± 2.29	b	55.25 ± 2.81	a	23.37 ± 0.41	b	51.01 ± 1.16	b	0.48 ± 0.04	b	226.98 ± 2.06		c	30.97 ± 1.92	a	65.87 ± 5.01	a	96.84 ± 5.63	a	715.5 ± 10.83			c
Rocket	557.84 ± 31.16	a	2.46 ± 0.12	b	43.18 ± 3.71	a	75.62 ± 1.75	a	1.69 ± 0.37	b	680.78 ± 34.68		b	2.32 ± 0.17	b	n.d.		2.32 ± 0.17	c	1053.02 ± 50.89			b
0	252.42 ± 103.5	a	23.2 ± 10.14	a	38.5 ± 7.16	a	63.91 ± 6.16		316.55 ± 158.08	b	660.44 ± 110.91		a	18.35 ± 7.54		42.32 ± 11.58		40.45 ± 15.83	a	992.83 ± 80.41			b
6	220.44 ± 85.49	b	20.62 ± 9.08	b	33.8 ± 5.06	a	61.49 ± 4.72		384.14 ± 191.87	a	688.72 ± 134.57		a	14.68 ± 5.83		39.56 ± 12.58		36.16 ± 14.56	a	1053.38 ± 86.86			a
12	187.19 ± 67.9	c	16.4 ± 7.2	c	27.53 ± 1.8	b	64.54 ± 6.34		356.79 ± 177.63	a	621.75 ± 123.91		b	16.89 ± 6.31		41.7 ± 11.53		39.06 ± 14.75	a	960.26 ± 85.1			b
Lettuce,0	4.3 ± 0.19	e	3.11 ± 0.05	d	n.d.		n.d.		948.58 ± 16.38	c	955.99 ± 16.23		b	n.d.		16.46 ± 0.69		16.46 ± 0.69	b	1090.12 ± 14.6			c
Lettuce,6	5.5 ± 0.43	e	2.53 ± 0.1	d	n.d.		n.d.		1150.67 ± 32.89	a	1158.69 ± 32.69		a	n.d.		15.93 ± 0.51		15.93 ± 0.51	b	1335.39 ± 32.59			a
Lettuce,12	6.24 ± 0.19	e	1.87 ± 0.16	d	n.d.		n.d.		1066.65 ± 26.1	b	1074.77 ± 26.16		a	n.d.		17.13 ± 0.71		17.13 ± 0.71	b	1288.34 ± 22.52			ab
Mustard,0	90.0 ± 2	d	63.75 ± 0.89	a	22.74 ± 0.42	c	50.53 ± 3.11		0.39 ± 0.03	d	227.41 ± 4.03		f	34.78 ± 3.77		68.17 ± 1.19		102.95 ± 4.96	a	680.04 ± 8.94			e
Mustard,6	97.7 ± 2.84	d	56.87 ± 1.79	b	22.66 ± 0.67	c	51.42 ± 1.8		0.45 ± 0.03	d	229.1 ± 4.26		f	27.34 ± 3.13		63.18 ± 15.26		90.51 ± 16.67	a	740.51 ± 14.47			e
Mustard,12	102.9 ± 2.96	d	45.13 ± 1.53	c	24.72 ± 0.24	c	51.07 ± 1.71		0.61 ± 0.01	d	224.43 ± 3.28		f	30.78 ± 2.5		66.26 ± 7.81		97.04 ± 6.29	a	725.95 ± 11.04			e
Rocket,0	662.97 ± 17.56	a	2.75 ± 0.15	d	54.27 ± 2.79	a	77.28 ± 1.03		0.67 ± 0.03	d	797.94 ± 20.9		c	1.93 ± 0.03		n.d.		1.93 ± 0.03	b	1208.32 ± 20.66			b
Rocket,6	558.13 ± 5.98	b	2.45 ± 0.19	d	44.93 ± 1.97	b	71.57 ± 2.55		1.29 ± 0.09	d	678.37 ± 9.58		d	2.03 ± 0.04		n.d.		2.03 ± 0.04	b	1084.25 ± 9.16			c
Rocket,12	452.42 ± 14.93	c	2.18 ± 0.14	d	30.34 ± 2.86	c	78 ± 4.08		3.09 ± 0.21	d	566.03 ± 21.35		e	2.99 ± 0.09		n.d.		2.99 ± 0.09	b	866.48 ± 24.81			d

n.s., *, **, *** Nonsignificant or significant at *p* ≤ 0.05, 0.01, and 0.001, respectively. Different letters within each column indicate significant differences according to Tukey-Kramer HSD test (*p* = 0.05).

## Data Availability

The datasets generated for this study are available on request to the corresponding author.

## References

[1] Kyriacou M.C., Rouphael Y., Di Gioia F., Kyratzis A., Serio F., Renna M., De Pascale S., Santamaria P. (2016). Micro-scale vegetable production and the rise of microgreens. Trends Food Sci. Technol..

[2] Kyriacou M.C., Soteriou G.A., Colla G., Rouphael Y. (2019). The occurrence of nitrate and nitrite in Mediterranean fresh salad vegetables and its modulation by preharvest practices and postharvest conditions. Food Chem..

[3] Caracciolo F., El-Nakhel C., Raimondo M., Kyriacou M.C., Cembalo L., de Pascale S., Rouphael Y. (2020). Sensory attributes and consumer acceptability of 12 microgreens species. Agronomy.

[4] Verlinden S. (2020). Microgreens: Definitions, Product Types, and Production Practices. Horticultural Reviews.

[5] Palmitessa O.D., Renna M., Crupi P., Lovece A., Corbo F., Santamaria P. (2020). Yield and quality characteristics of brassica microgreens as affected by the NH4:NO3 molar ratio and strength of the nutrient solution. Foods.

[6] El-Nakhel C., Pannico A., Graziani G., Kyriacou M.C., Gaspari A., Ritieni A., De Pascale S., Rouphael Y. (2021). Nutrient Supplementation Configures the Bioactive Profile and Production Characteristics of Three Brassica L. Microgreens Species Grown in Peat-Based Media. Agronomy.

[7] Teng J., Liao P., Wang M. (2021). The role of emerging micro-scale vegetables in human diet and health benefits—An updated review based on microgreens. Food Funct..

[8] Paradiso V.M., Castellino M., Renna M., Santamaria P., Caponio F. (2020). Setup of an extraction method for the analysis of carotenoids in microgreens. Foods.

[9] El-Nakhel C., Pannico A., Kyriacou M.C., Giordano M., De Pascale S., Rouphael Y. (2019). Macronutrient deprivation eustress elicits differential secondary metabolites in red and green-pigmented butterhead lettuce grown in a closed soilless system. J. Sci. Food Agric..

[10] Bian Z., Wang Y., Zhang X., Li T., Grundy S., Yang Q., Cheng R. (2020). A review of environment effects on nitrate accumulation in leafy vegetables grown in controlled environments. Foods.

[11] Simanavičius L., Viršile A. (2018). The effects of led lighting on nitrates, nitrites and organic acids in tatsoi. Res. Rural Dev..

[12] European Food Safety Agency (EFSA) (2008). Opinion of the Scientific Panel on Contaminants in the Food chain on a request from the European Commission to perform a scientific risk assessment on nitrate in vegetables. EFSA J..

[13] Cavaiuolo M., Ferrante A. (2014). Nitrates and glucosinolates as strong determinants of the nutritional quality in rocket leafy salads. Nutrients.

[14] (2006). European Commission Regulation (EC) No 1882/2006 of 19 December 2006 laying down methods of sampling and analysis for the official control of the levels of nitrates in certain foodstuffs. Off. J. Eur. Union.

[15] Anjana S.U., Iqbal M. (2007). Nitrate accumulation in plants, factors affecting the process, and human health implications. A review. Agron. Sustain. Dev..

[16] Colla G., Kim H.J., Kyriacou M.C., Rouphael Y. (2018). Nitrate in fruits and vegetables. Sci. Hortic..

[17] Kyriacou M.C., El-Nakhel C., Graziani G., Pannico A., Soteriou G.A., Giordano M., Ritieni A., De Pascale S., Rouphael Y. (2019). Functional quality in novel food sources: Genotypic variation in the nutritive and phytochemical composition of thirteen microgreens species. Food Chem..

[18] Lenzi A., Orlandini A., Bulgari R., Ferrante A., Bruschi P. (2019). Antioxidant and mineral composition of three wild leafy species: A comparison between microgreens and baby greens. Foods.

[19] Pannico A., Graziani G., El-Nakhel C., Giordano M., Ritieni A., Kyriacou M.C., Rouphael Y. (2020). Nutritional stress suppresses nitrate content and positively impacts ascorbic acid concentration and phenolic acids profile of lettuce microgreens. Italus Hortus.

[20] Liu H., Kang Y., Zhao X., Liu Y., Zhang X., Zhang S. (2019). Effects of elicitation on bioactive compounds and biological activities of sprouts. J. Funct. Foods.

[21] Galieni A., Falcinelli B., Stagnari F., Datti A., Benincasa P. (2020). Sprouts and microgreens: Trends, opportunities, and horizons for novel research. Agronomy.

[22] Onakpoya I.J., Spencer E.A., Thompson M.J., Heneghan C.J. (2015). The effect of chlorogenic acid on blood pressure: A systematic review and meta-analysis of randomized clinical trials. J. Hum. Hypertens..

[23] Tajik N., Tajik M., Mack I., Enck P. (2017). The potential effects of chlorogenic acid, the main phenolic components in coffee, on health: A comprehensive review of the literature. Eur. J. Nutr..

[24] Whitaker B.D., Stommel J.R. (2003). Distribution of hydroxycinnamic acid conjugates in fruit of commercial eggplant (Solanum melongena L.) cultivars. J. Agric. Food Chem..

[25] Santos J.S., Escher G.B., Vieira do Carmo M., Azevedo L., Boscacci Marques M., Daguer H., Molognoni L., Inés Genovese M., Wen M., Zhang L. (2020). A new analytical concept based on chemistry and toxicology for herbal extracts analysis: From phenolic composition to bioactivity. Food Res. Int..

[26] Lesjak M., Beara I., Simin N., Pintać D., Majkić T., Bekvalac K., Orčić D., Mimica-Dukić N. (2018). Antioxidant and anti-inflammatory activities of quercetin and its derivatives. J. Funct. Foods.

[27] Terao J., Yamaguchi S., Shirai M., Miyoshi M., Moon J.H., Oshima S., Inakuma T., Tsushida T., Kato Y. (2001). Protection by Quercetin and Quercetin 3-O-β-D-glucuronide of peroxynitrite-induced antioxidant consumption in human plasma low-density lipoprotein. Free Radic. Res..

[28] Kyriacou M.C., El-Nakhel C., Pannico A., Graziani G., Soteriou G.A., Giordano M., Zarrelli A., Ritieni A., De Pascale S., Rouphael Y. (2019). Genotype-Specific Modulatory Effects of Select Spectral Bandwidths on the Nutritive and Phytochemical Composition of Microgreens. Front. Plant Sci..

[29] Rouphael Y., Colla G., Giordano M., El-Nakhel C., Kyriacou M.C., De Pascale S. (2017). Foliar applications of a legume-derived protein hydrolysate elicit dose-dependent increases of growth, leaf mineral composition, yield and fruit quality in two greenhouse tomato cultivars. Sci. Hortic..

[30] Murphy C.J., Llort K.F., Pill W.G. (2010). Factors affecting the growth of microgreen table beet. Int. J. Veg. Sci..

[31] Rouphael Y., Kyriacou M.C., Petropoulos S.A., De Pascale S., Colla G. (2018). Improving vegetable quality in controlled environments. Sci. Hortic..

[32] Pathare P.B., Opara U.L., Al-Said F.A.-J. (2013). Colour Measurement and Analysis in Fresh and Processed Foods: A Review. Food Bioprocess Technol..

[33] Kyriacou M.C., Rouphael Y. (2018). Towards a new definition of quality for fresh fruits and vegetables. Sci. Hortic..

[34] Brazaityte A., Sakalauskiene S., Samuoliene G., Jankauskiene J., Viršile A., Novičkovas A., Sirtautas R., Miliauskiene J., Vaštakaite V., Dabašinskas L. (2015). The effects of LED illumination spectra and intensity on carotenoid content in Brassicaceae microgreens. Food Chem..

[35] Di Gioia F., De Bellis P., Mininni C., Santamaria P., Serio F. (2017). Physicochemical, agronomical and microbiological evaluation of alternative growing media for the production of rapini (*Brassica rapa* L.) microgreens. J. Sci. Food Agric..

[36] Kyriacou M.C., El-Nakhel C., Pannico A., Graziani G., Soteriou G.A., Giordano M., Palladino M., Ritieni A., De Pascale S., Rouphael Y. (2020). Phenolic constitution, phytochemical and macronutrient content in three species of microgreens as modulated by natural fiber and synthetic substrates. Antioxidants.

[37] El-Nakhel C., Pannico A., Graziani G., Kyriacou M.C., Giordano M., Ritieni A., De Pascale S., Rouphael Y. (2020). Variation in macronutrient content, phytochemical constitution and in vitro antioxidant capacity of green and red butterhead lettuce dictated by different developmental stages of harvest maturity. Antioxidants.

[38] Ferrón-Carrillo F., Guil-Guerrero J.L., González-Fernández M.J., Lyashenko S., Battafarano F., da Cunha-Chiamolera T.P.L., Urrestarazu M. (2021). LED Enhances Plant Performance and Both Carotenoids and Nitrates Profiles in Lettuce. Plant Foods Hum. Nutr..

[39] Pinto E., Almeida A.A., Aguiar A.A., Ferreira I.M.P.L.V.O. (2015). Comparison between the mineral profile and nitrate content of microgreens and mature lettuces. J. Food Compos. Anal..

[40] Borgognone D., Rouphael Y., Cardarelli M., Lucini L., Colla G. (2016). Changes in biomass, mineral composition, and quality of cardoon in response to NO^3−^:Cl^−^ ratio and nitrate deprivation from the nutrient solution. Front. Plant Sci..

[41] Gharibzahedi S.M.T., Jafari S.M. (2017). The importance of minerals in human nutrition: Bioavailability, food fortification, processing effects and nanoencapsulation. Trends Food Sci. Technol..

[42] Levander O.A. (1990). Fruit and Vegetable Contributions to Dietary Mineral Intake in Human Health and Disease. HortScience.

[43] Xiao Z., Codling E.E., Luo Y., Nou X., Lester G.E., Wang Q. (2016). Microgreens of Brassicaceae: Mineral composition and content of 30 varieties. J. Food Compos. Anal..

[44] Neugart S., Baldermann S., Hanschen F.S., Klopsch R., Wiesner-Reinhold M., Schreiner M. (2018). The intrinsic quality of brassicaceous vegetables: How secondary plant metabolites are affected by genetic, environmental, and agronomic factors. Sci. Hortic..

[45] Young A.J., Lowe G.M. (2001). Antioxidant and prooxidant properties of carotenoids. Arch. Biochem. Biophys..

[46] Kvansakul J., Rodriguez-Carmona M., Edgar D.F., Barker F.M., Köpcke W., Schalch W., Barbur J.L. (2006). Supplementation with the carotenoids lutein or zeaxanthin improves human visual performance. Ophthalmic Physiol. Opt..

[47] Xiao Z., Lester G.E., Luo Y., Wang Q. (2012). Assessment of Vitamin and Carotenoid Concentrations of Emerging Food Products: Edible Microgreens. J. Agric. Food Chem..

[48] De la Fuente B., López-García G., Máñez V., Alegría A., Barberá R., Cilla A. (2019). Evaluation of the bioaccessibility of antioxidant bioactive compounds and minerals of four genotypes of Brassicaceae microgreens. Foods.

[49] Klopsch R., Baldermann S., Hanschen F.S., Voss A., Rohn S., Schreiner M., Neugart S. (2019). Brassica-enriched wheat bread: Unraveling the impact of ontogeny and breadmaking on bioactive secondary plant metabolites of pak choi and kale. Food Chem..

[50] Sun J., Xiao Z., Lin L.Z., Lester G.E., Wang Q., Harnly J.M., Chen P. (2013). Profiling polyphenols in five brassica species microgreens by UHPLC-PDA-ESI/HRMSn. J. Agric. Food Chem..

[51] Alfaifi M., Alsayari A., Gurusamy N., Louis J., Elbehairi S.E., Venkatesan K., Annadurai S., Asiri Y.I., Shati A., Saleh K. (2020). Analgesic, Anti-Inflammatory, Cytotoxic Activity Screening and UPLC-PDA-ESI-MS Metabolites Determination of Bioactive Fractions of Kleinia pendula. Molecules.

[52] Granato D., Shahidi F., Wrolstad R., Kilmartin P., Melton L.D., Hidalgo F.J., Miyashita K., van Camp J., Alasalvar C., Ismail A.B. (2018). Antioxidant activity, total phenolics and flavonoids contents: Should we ban in vitro screening methods?. Food Chem..

